# Fatigue assessment in patients with rheumatoid arthritis

**DOI:** 10.1192/j.eurpsy.2026.11228

**Published:** 2026-07-31

**Authors:** A. Feki, I. Sallemi, A. Abbes, Z. Gassara, H. Fourati

**Affiliations:** 1 Rheumatology Department; 2 Occupational Medicine Department, Hedi Chaker Hospital, Sfax, Tunisia

## Abstract

**Introduction:**

Rheumatoid arthritis (RA) is a chronic inflammatory autoimmune disease that affects joints and causes pain, swelling and stiffness. Fatigue is a major and often underestimated symptom of this disease, which can be intense, persistent and debilitating. This fatigue has a significant impact on patients’ quality of life, limiting their daily and social abilities, and can be considered one of the most serious and disabling symptoms of the disease.

**Objectives:**

To assess fatigue in patients with rheumatoid arthritis and to explore associated factors.

**Methods:**

We conducted a descriptive and analytic study. We opted for a non-random sampling method. The population consisted of 45 patients with RA followed up at a rheumatology department. We used the Bristol Rheumatoid Arthritis Fatigue Multi-Dimensional Questionnaire (BRAF-MDQ), which assesses total, physical, coping, cognitive and emotional fatigue. Data confidentiality and participant anonymity were respected.

**Results:**

This study showed that the majority of the population was aged between 41 and 60 years, with a notable female predominance of 84.4%. The results of the assessment of fatigue according to the four dimensions (physical, coping, cognitive and emotional) showed that 53.3% of our population experienced moderate fatigue, 26.7% severe fatigue and 20% mild fatigue. Cross-tabulation revealed moderate to severe fatigue, with a significant association between the overall fatigue score and several variables: gender, level of education, socioeconomic status, and living environment, as well as DAS28 (p<0.05).

**Conclusions:**

Chronic fatigue associated with rheumatoid arthritis significantly impairs patients’ quality of life. Often overlooked, it affects patients physically, emotionally and socially.

**Disclosure of Interest:**

None Declared

